# Laboratory diagnosed microbial infection in English UK Biobank participants in comparison to the general population

**DOI:** 10.1038/s41598-022-20635-4

**Published:** 2023-01-10

**Authors:** Bridget Hilton, Daniel J. Wilson, Anne-Marie O’Connell, Dean Ironmonger, Justine K. Rudkin, Naomi Allen, Isabel Oliver, David H. Wyllie

**Affiliations:** 1 UK Health Security Agency, London, UK; 2 Nuffield Department of Population Health, Big Data Institute, Li Ka Shing Centre for Health Information and Discovery, University of Oxford, Oxford, UK; 3 Nuffield Department of Medicine, University of Oxford, Oxford, UK

**Keywords:** Microbiology, Infectious-disease epidemiology

## Abstract

Understanding the genetic and environmental risk factors for serious bacterial infections in ageing populations remains incomplete. Utilising the UK Biobank (UKB), a prospective cohort study of 500,000 adults aged 40–69 years at recruitment (2006–2010), can help address this. Partial implementation of such a system helped groups around the world make rapid progress understanding risk factors for SARS-CoV-2 infection and COVID-19, with insights appearing as early as May 2020. In principle, such approaches could also to be used for bacterial isolations. Here we report feasibility testing of linking an England-wide dataset of microbial reporting to UKB participants, to enable characterisation of microbial infections within the UKB Cohort. These records pertain mainly to bacterial isolations; SARS-CoV-2 isolations were not included. Microbiological infections occurring in patients in England, as recorded in the Public Health England second generation surveillance system (SGSS), were linked to UKB participants using pseudonymised identifiers. By January 2015, ascertainment of laboratory reports from UKB participants by SGSS was estimated at 98%. 4.5% of English UKB participants had a positive microbiological isolate in 2015. Half of UKB isolates came from 12 laboratories, and 70% from 21 laboratories. Incidence rate ratios for microbial isolation, which is indicative of serious infection, from the UKB cohort relative to the comparably aged general population ranged from 0.6 to 1, compatible with the previously described healthy participant bias in UKB. Data on microbial isolations can be linked to UKB participants from January 2015 onwards. This linked data would offer new opportunities for research into the role of bacterial agents on health and disease in middle to-old age.

## Introduction

This paper concerns an approach to understanding the determinants and sequelae of infection in older humans, by using two existing data sources in England. This is of interest because bacterial infection is an important cause of death in older individuals^1^.

### Studying bacterial infection in older individuals

Incidence rates of bacterial infections increase markedly with age. For example, English surveillance data shows that the incidence of *E. coli* bacteraemia is more than tenfold higher in 45–64 year old men, and about 100-fold higher in over 75 year olds^2^ compared with 15–44 year olds. Similar trends are observed with *S. aureus*, *S. pyogenes* and *S. pneumoniae* bacteraemia^3,4^. The age-associated increased incidence of severe infection is observed both in individuals with a history of hospitalisation and in individuals without prior hospital exposure^5^. Marked age-associated increases in infection rates are also observed in community-origin conditions diagnosed syndromically in general practice, such as respiratory infections^6^.

The reasons for the age-related increase in infection incidence are not fully understood. Possible contributing factors include environmental risk factors, including housing, nutrition and other aspects of lifestyle. There is well documented population variability in innate immune function, e.g. in baseline inflammatory activity as reflected in serum CRP concentrations^7^ which may be relevant to individual infection risk. Age-specific declines in adaptive immunity (e.g. T cell responses, antibody concentrations) may also be relevant, and have been shown, using sero-epidemiological and vaccination studies, to be related to the age-related increase in pneumococcal pneumonia^8^ and herpes zoster infection^9^. Finally, germline genetic polymorphisms that predispose to infection^10–12^ may be revealed, as biological predispositions increase.

### The advantages of cohort studies

Assessing the impact of environmental, innate and adaptive immune, and genetic risk factors for infection in older adults is complex. For the comprehensive and reliable quantification of the combined effects of lifestyle, environment, genes and other exposures on a range of infectious diseases, prospective studies have a number of important advantages over retrospective case–control studies:

- They allow a wide range of different infectious diseases to be studied.
- Exposures can be assessed prior to disease development, which usually improves detail and accuracy (reduced variance) of information. Causal interpretations of associations between prior exposures and subsequent outcomes may be more robust (or enriched for true positives) because the temporality of exposure-disease outcomes are well defined, compared to other retrospective case–control studies.
- Investigation becomes possible into disease risk factors that might be affected by infection and/or its treatments (e.g., immune status blood marker concentrations, gut microbiome/metabolomics) or by an individual’s response to developing a bacterial infection (e.g., weight, physical activity, diet, host genetics).
- Prospective studies are also able to better assess severe infections that have a high case-fatality rate, as such cases cannot readily be studied retrospectively.
- Prospective studies enable research into how infectious disease ‘exposure’ is related to a wide range of subsequent chronic health conditions.

However, because the incidence of severe infection in the general population is low (*E. coli* blood stream infection, which is the commonest blood stream infection in the UK, has an incidence of about 50 per 100,000 in 45–64 year-olds)^2^, then prospective studies investigating infection risk factors in the general population need to be large.

Here, we discuss (i) how infection is ascertained via microbiological testing; (ii) the recording of microbiological information in a central database in England; and (iii) how linkage of this database to a large-scale population cohort, such as UK Biobank, will enable novel research into the determinants of bacterial infection and its role in subsequent health and disease.

### The diagnosis of infection

Infection can be diagnosed based on clinical presentation, although symptoms are less predictive of bacterial infections in older individuals^1^. The addition of radiological imaging (e.g. chest X-rays) can provide indication of infection^13,14^, but radiological diagnosis is unusual in primary care, at least in the UK. Microbiological sampling can also contribute to the diagnosis of infection and reveal the causative organism: a diagnosis of severe bacterial infection can be made following microbiological culture of normally sterile sites, including blood, peritoneal fluid, and cerebrospinal fluid^15,16^. Positive microbiological cultures of urine are also frequently associated with clinical infection, although microbiological isolation without apparent illness is common in individuals with catheters, and in some elderly individuals^17,18^.

### The second generation surveillance system (SGSS)

In England, the processing of microbiological specimens predominantly occurs in hospital laboratories run by the National Health Service. As part of its role in monitoring and improving population health, Public Health England (PHE; succeeded by the UK Health Security Agency, UKHSA) established a database (SGSS) that includes details of all positive microbiological isolates (microbial cultures) on which antimicrobial susceptibility testing is performed in all NHS Trusts in England. Near universal coverage was achieved by 2015, following work undertaken in support of the UK Government’s Antimicrobial Resistance Strategy^19^. The database is a cornerstone of PHE surveillance, with aggregated information on bacterium/resistance profile combinations (‘bug-drug combinations’)^19^ being fed back to the healthcare system via a web-based portal in an effort to tailor prescribing to local resistance patterns.

### The UK Biobank

**Figure 1:**
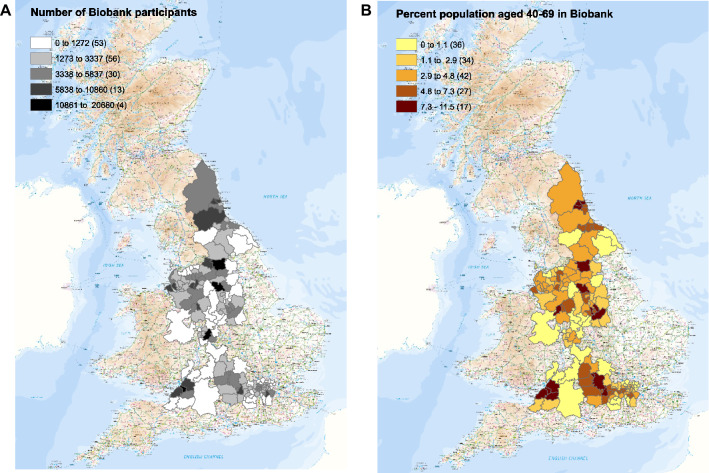
The numbers of UK Biobank participants identified in each local authority in which recruitment occurred (left), and the percentage of the population in each local authority recruited (right). Maps were made with ARCGIS by UKHSA.

The UK Biobank (UKB) is a prospective cohort study, which recruited around 500,000 men and women aged 40–69 years who lived within travelling distance of one of 22 recruitment centres between 2006 and 2010^20^. It was designed to assess the genetic and environmental determinants that contribute to common life-threatening and disabling diseases^20^. Of the 500,000 participants recruited to UK Biobank, 445,023 (89%) were resident in major population areas across England and represent a sociodemographically heterogeneous group (Fig. 1).

In addition to providing baseline health data and biological samples for biomarker measurement and genotyping, participants continue to be invited to undertake ongoing enhancements (e.g., multi-modal imaging, physical activity monitoring, completion of a series of web-based questionnaires). All participants also consented to linkage of their health records, such as death, cancer, hospital inpatient records and primary care data^21^. Therefore, the UK Biobank cohort is one potential setting in which study of the determinants of microbial infection and of the sequelae of infection (including death) can be carried out, contingent on linking UK Biobank participants to laboratory records of microbial isolation.

### Objective of this work

Here, we report the results of a pilot project linking data from UKB to the microbial isolations reported in SGSS. The objectives of the study were:

(i) To assess technical feasibility of the approach.
(ii) To describe patterns of bacterial infection in the UKB cohort compared with the general population.
(iii) To assess the extent of any ‘healthy patient effect’ (UKB participants being more healthy than the general population, as assessed by microbial isolation rates).

## Results

### NHS numbers are sufficient for data linkage of microbiology samples in England

**Figure 2:**
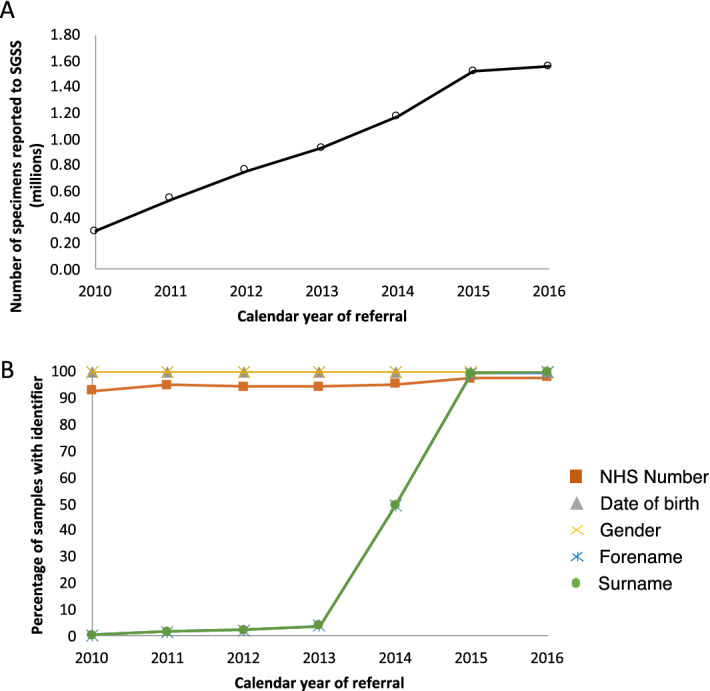
The numbers of specimens reported to SGSS (**A**) and the percentage of those specimens with various identifiers (**B**).

Between 1 April 2010 and 30 June 2016, data on microbial isolates from 4,726,417 samples (in 4,066,974 individuals) aged 40–69 years were deposited within SGSS (Fig. S1, Fig. 2A). The majority of samples had NHS number (exceeding 90% across all years of analysis, but increasing to over 98% from 2015 onwards, because using NHS numbers in laboratory test requests was mandated by statute in 2014). A change in the nature of the data feeds into SGSS during 2014 also led to a marked increase in availability of surnames from 2015 onwards (Fig. 2B). We considered whether linkage using additional identifiers (surname, forename, date birth) increased the proportion of linked records (see “Methods” section); investigations indicated that use of composite identifiers (in addition to NHS numbers) were likely to link records from individuals other than the intended individual. Given the almost universal nature of NHS number use post 2015, and the statutory requirement that the NHS uses it going forward, we conducted subsequent record linkage using NHS number alone.

### Near complete coverage of English UKB microbiological isolation by 2015

Numbers of samples arriving in SGSS increased year-on-year 2010 to 2015, before stabilising (Fig. 2A). This increase coincided with a PHE initiative to encourage and assist all laboratories in England to report routinely to SGSS; by 2015, only three out of 172 laboratories were not doing so.

Based on the proportion of laboratories reporting to SGSS, the local authority areas in England they cover, and the residence of UK Biobank participants, the estimated ascertainment (i.e., recording in SGSS) of positive microbiological samples for UK Biobank participants from 2015 onwards is 98%. However, prior to 2015 the reporting rates to SGSS varied markedly between local authorities.

### Patterns of microbiological isolation in UKB participants

During 2015 (the year from which coverage for UKB participants in England can be considered almost complete), 21,361 individuals, corresponding to 4.80% of UK Biobank participants in England, had a positive microbiological culture recorded in SGSS. As expected from national data^2–4,22^ *E. coli* (isolated from 1.93% of the cohort) and *S. aureus* (isolated from 0.67% of the cohort) were the commonest isolates, but major community and hospital associated pathogens were also represented, including *Enterococcus* spp.*, Ps. aeruginosa*, various *Enterobacteriaceae* and *Streptococcus* spp. (Table 1).

**Table 1:** The number of UK Biobank participants with microbial isolations recorded in SGSS in 2015 for the 25 most common organisms isolated.

Organism	Number of English UK Biobank subjects with isolation recorded in SGSS in 2015	% of English Biobank subjects with microbiological isolations in 2015
*Escherichia coli*	8584	1.93
*Staphylococcus aureus*	2968	0.67
Enterobacteraciae, not speciated	2271	0.51
*Haemophilus influenzae*	1031	0.23
Enterococcus sp.	826	0.19
*Pseudomonas aeruginosa*	719	0.16
*Klebsiella pneumoniae*	527	0.12
Staphylococcus coagulase negative	507	0.11
Streptococcus group B	480	0.11
*Pseudomonas* sp.	368	0.08
*Proteus* sp.	345	0.08
*Enterococcus faecalis*	320	0.07
Streptococcus group A	318	0.07
*Proteus mirabilis*	296	0.07
*Moraxella catarrhalis*	288	0.06
*Streptococcus pneumoniae*	271	0.06
*Campylobacter* sp.	241	0.05
*Enterobacter cloacae*	155	0.03
Streptococcus group G	148	0.03
*Citrobacter diversus* (*C. koseri*)	140	0.03
*Klebsiella oxytoca*	132	0.03
Staphylococcus other named	128	0.03
Streptococcus group C	122	0.03
*Klebsiella* sp.	107	0.02
*Enterococcus faecium*	69	0.02

Note that one individual’s cultures can yield more than one different microbe.

Isolates were derived from urinary, skin, sputum, blood, genital, and faecal samples, which are typical of current microbiological usage in NHS microbiology laboratories (Table 2). Urinary isolates were most common, with 12,468 individuals (2.80% of the English UK Biobank cohort) having a positive urinary isolate in 2015. By contrast, individuals with any positive blood cultures were relatively uncommon, with only 701 individuals with such isolates (0.16% of the cohort).

**Table 2:** The number of UK Biobank participants with samples recorded in SGSS in 2015, stratified by sample type from which the isolate was obtained.

Specimen type	Number of UK Biobank subjects with isolations from Specimen types recorded in SGSS in 2015	% of English Biobank subjects with microbiological isolations in 2015
Urine/kidney	12468	2.80
Skin/wound	2534	0.57
Sputum	1808	0.41
Blood	701	0.16
Swab	649	0.15
Lower genital tract	533	0.12
Faeces/lower gastro-intestinal tract	433	0.10
Nose	413	0.09
Pus source unknown	242	0.05
Tissue	181	0.04
Middle ear/mastoid	190	0.04
Lower respiratory tract	131	0.03
Unknown	147	0.03
Fluid—nos	122	0.03
Throat	92	0.02
Eye	74	0.02
Intra-vascular line	44	0.01
Upper respiratory tract	40	0.01

27% of the microbiological isolates from UK Biobank were recovered by five microbiology laboratories, 52% by twelve microbiology laboratories, and 70% by 21 laboratories (Table S1), which is relevant when considering the resource implications of obtaining microbial isolate specimens from UKB participants prospectively. Complete coverage by such a program would require participation of a large number of laboratories: 121 microbiology laboratories reported at least one specimen from a UK Biobank subject in 2015, 41 reported more than 100 specimens, 22 reported more than 300, while ten reported more than 600 specimens.

### Healthy participant effect

To qualify any possible “healthy participant” effect in infection outcomes, we analysed the last five quarters of the study period, as we considered the data most complete during this period (Fig. 2). Table 3 shows isolation results for *E. coli, S. pneumoniae, S. aureus,* and *Campylobacter,* which are the most commonly isolated pathogens of urinary, respiratory, skin/wound, and bowel infection, respectively^2,3,5,22–24^, as well as *Salmonella*, which is a rare isolate. Of these organisms, *E. coli* and *S. aureus* were by far the most common infections identified, with the majority isolated in primary care (Table 3).

**Table 3:** Numbers of individuals and (in brackets) rate per 1000 persons years observed with isolation of various microorganisms.

Population	Facility	Gender	*E. coli*	*S. pneumoniae*	*S. aureus*	*Campylo-bacter*	*Salmonella*
Most commonly infected site			Urine	Respiratory	Skin/wound	Bowel	Bowel
Gen. pop.	Acute	F	201,267 (19.3)	4227 (0.4)	107,600 (10.3)	2183 (0.2)	584 (0.1)
**UKB**	**Acute**	**F**	3820 (14.0)	106 (0.4)	1653 (6.0)	51 (0.2)	17 (0.1)
Gen. pop.	Acute	M	66,402 (6.6)	5248 (0.5)	154,101 (15.2)	2953 (0.3)	618 (0.1)
**UKB**	**Acute**	**M**	1173 (5.1)	88 (0.5)	2224 (9.7)	57 (0.2)	12 (0.1)
Gen. pop.	Comm/GP	F	394,530 (37.9)	5244 (0.5)	84,131 (8.1)	4733 (0.5)	757 (0.1)
**UKB**	**Comm/GP**	**F**	10,647 (38.9)	105 (0.4)	1339 (4.9)	162 (0.6)	22 (0.1)
Gen. pop.	Comm/GP	M	82,836 (8.2)	4880 (0.5)	90,481 (8.9)	5817 (0.6)	693 (0.1)
**UKB**	**Comm/GP**	**M**	2007 (8.8)	114 (0.5)	1518 (6.6)	205 (0.9)	24 (0.1)

Numbers are presented stratified. Population refers to either the general population aged 40–69 during UK Biobank recruitment (Gen. Pop.), where the specimen was sent from (Facility = either Acute NHS Trusts, which contain inpatients (Acute) or from out of hospital settings (Community/General Practice (Comm/GP)).

Overall, rates of microbial isolation from UK Biobank participants are of similar order and pattern to those seen in the general population (Tables 3, 4). As expected, there is a higher rate of isolation of *E. coli* in women than in men (e.g., for samples sent from general practice settings, isolate rate was 38.9 in females vs. 8.8 in men per 1000 person years observation), while *S. aureus* displays the opposite pattern (4.9 vs. 6.6 per 1000 person years isolation, in women and men, respectively). For all *E. coli* isolated from primary care, overall isolation rates are similar in UKB participants and in the general population (Tables 3, 4). In contrast, rates of *S. pneumoniae* and *S. aureus* isolation, and isolation of resistant *E. coli*, are slightly lower in UKB populations than in the general population, with incidence rate ratio estimates of 0.6 to 0.8 in different groups (Tables 3, 4). This effect is also seen in resistant *E. coli* isolates from primary care (Tables 3, 4).

**Table 4:** Incidence rate ratios (IRR, and 95% confidence intervals) comparing isolation of *E. coli* (including resistant *E. coli* populations), *S. pneumoniae* and *S. aureus* from microorganisms recorded in SGSS April 2015 to August 2016, stratified by UK Biobank status.

Facility	Gender	*E. coli*		*E. coli,* resistant to 3rd gen. cephalosporins		*E. coli,* resistant to ciprofloxacin		*S. pneumoniae*		*S. aureus*	
		IRR	95% CI	IRR	95% CI	IRR	95% CI	IRR	95% CI	IRR	95% CI
Acute	F	0.72	0.70, 0.75	0.94	0.91, 0.97	0.84	0.56, 0.6	0.96	0.78, 1.16	0.59	0.56, 0.61
Acute	M	0.78	0.74, 0.83	0.73	0.70, 0.77	0.84	0.80, 0.87	0.74	0.59, 0.91	0.64	0.61, 0.67
Comm/GP	F	1.03	1.01, 1.05	0.74	0.72, 0.75	0.82	0.81, 0.84	0.76	0.62, 0.93	0.61	0.57, 0.64
Comm/GP	M	1.07	1.02, 1.12	0.78	0.75, 0.82	0.83	0.79, 0.86	1.03	0.85, 1.24	0.74	0.70, 0.78

Microbiological isolations occur both in hospital samples (Facility = ‘Acute’) and out of hospital community settings/general practice (Facility = ‘Comm/GP’). Populations at risk of isolation differ between these populations: we used UK Biobank subjects as the denominator for the UK Biobank subjects, and the number of individuals 45–69 in mid 2015 as a denominator for non-UK Biobank cases.

The data support the idea that UK Biobank subjects are healthier than the general population, although the estimated ‘healthy patient effect’ differs somewhat between organisms.

## Discussion

We have demonstrated the feasibility of linking prospective cohort data (i.e., UK Biobank) with a national dataset containing information on microbial isolates in England (SGSS). This expands the scope of a previously published approach that links SARS-CoV-2 test results in UKB^26^ to a far greater spectrum of infectious diseases. The initiative was assisted by a clear ethical framework and common data model within both data sources, use of a proven pseudonymisation technology^11^, and high personal identifier quality and utilisation (NHS numbers) in SGSS. The SGSS dataset has near complete (> 98%) coverage of England from 2015 onwards and represents a single dataset containing microbial isolates from both primary and secondary care sources. It includes a data feed including all organisms on which antimicrobial susceptibility testing was performed, and the susceptibility results obtained. As microbiological standard operating procedures require that antimicrobial susceptibility testing be performed on clinically significant microbiological isolates^25^, SGSS is likely to include a very high proportion of significant bacterial isolations in England.

Integration of microbiological data held by PHE (now the UK Health Security Agency) with the UK Biobank study, the feasibility of which we have demonstrated here, has a number of potential advantages for public health and biomedical research, many recently exemplified by studies of COVID-19^26–32^. Although microbiological data obtained before 2015 is available in SGSS only in some areas of England, which reduces ascertainment by SGSS of isolation from UK Biobank participants in the period 2010–2015, at the time of writing, eight full years (2015–2022) of nearly complete data can be linked, representing a large and powerful data source for epidemiological analysis.

However, before addressing these opportunities, we set out the limitations of the record linkage as implemented in this work.

Firstly, the UK Biobank cohort is derived from, but not necessarily representative of, the English population^33^. For example, as with almost all volunteer-based studies, those individuals who joined the study were slightly more healthy and wealthy than the sampling frame of the general UK population^33^. Compatible with the “healthy volunteer effect”, microbial isolation rates were generally lower in UK Biobank participants than in the similarly aged population resident in the same area. For example, incidence rate ratios for *S. aureus, S. pneumoniae,* and *E. coli* resistant to ciprofloxacin in UK Biobank participants relative to similarly aged women attending general practitioners in the same area were 0.61 (95% CI 0.57, 0.64), 0.76 (95% CI 0.62, 0.93), and 0.82 (0.81, 0.84), respectively. This likely reflects systematic differences in health care usage (such as use and selection of antibiotics) between UK Biobank and other subjects. However, such biases can be quantified and used to make valid assessments of exposure-disease associations that are generalizable to the wider population, despite its lack of representativeness^33^.

Secondly, the validity of the data received by SGSS depends on the microbiological processing of the samples occurring in multiple labs. SGSS performs standardisation of nomenclature received from these diverse laboratories (term-mapping to a standard ontology). Across England there exists some heterogeneity between protocols and platform technologies used in different microbiological laboratories. This persists despite efforts to standardise practice, including the Standards for Microbiological Investigation which have been published and widely adopted in the UK^25^, together with mandatory participation in external quality assurance schemes and periodic re-accreditation of laboratories. Therefore, some variation in results (for example, in antimicrobial susceptibility testing results, consequent on different methodologies being used) will exist across laboratories.

Thirdly, ascertainment of microbiological infection depends on access to medical care where relevant specimens are taken. Sampling policies may vary by medical practitioner^34^ At present, SGSS only records the origin (sampling location) and results of positive microbiological samples, so the overall quantity of samples taken cannot be computed directly from SGSS data. In theory, comparison with data within UK Biobank derived from GP clinical systems (some of which now receive electronic copies of microbiology results, both positive and negative) might address this, as well as providing a route to cross-validate information derived from both GP records and SGSS. A further factor influencing ascertainment concerns laboratory processing. Infrequently, laboratories change their processing methodology, for example when automated systems become available and these may be associated with ‘step changes’ in sensitivity of detection of pathogens. Monitoring the technologies used across UK Diagnostics laboratories, and the extent of microbial sampling with the UK Biobank cohort is an area for future work.

Similarly, even if the sampling location is known (i.e. hospital vs. community), for epidemiological purposes it may be relevant to attribute this sample to a hospital stay. It is not clear from microbiological reports alone whether a sample is from a patient who has recently been discharged from hospital. This can be achieved by linkage with hospital inpatient data, which is collated in UK Biobank, but which was not available in this present analysis.

Finally, most infection is diagnosed based on symptoms and signs, without any contribution from microbiological investigation. Consequently, it is predominantly syndromic presentations which are coded and recorded in electronic record systems in primary and sometimes secondary care. Such diagnosis becomes much less accurate in older individuals^1^. Compared with syndromic diagnosis, positive microbiological cultures from normally sterile sites have very high specificity^15,16,35^, with isolation recognised pathogens being indicative of severe infection in essentially all cases. By contrast, blood stream microbial isolation has a relatively low sensitivity for infection, particularly in mild disease and in respiratory infections, in which bacteraemia is detected in fewer than 20% of cases^36^. The specificity of microbial isolation from urine, which is much more common than isolation from blood cultures, is lower unless compatible symptoms are present^17,18^. It is high specificity which underlies the use of isolation of pathogens from blood cultures in infection surveillance programs^15,16,35^. Therefore, diagnoses of infection in the UK Biobank cohort using microbiological endpoints will allow specific identification of severe infections with a range of common organisms.

Nevertheless, the technical feasibility of monitoring microbiological isolations from circa 450,000 individuals offers a number of important opportunities.

Firstly, because the UK Biobank contains genomic data on all of its participants, host genetic variability can be investigated as risk factors for microbial isolation for a wide range of organisms (Supplementary Table S1). For example, in the context of COVID-19, a dynamic linkage approach based on the system described here was expedited for immediate deployment and contributed to analyses that revealed more than a dozen regions of the human genome involved in susceptibility to SARS-CoV-2 infection and severe COVID-19 (i.e. that required hospitalisation). It is likely that many host determinants of infection remain to be determined for the large number of non-COVID-19 infectious diseases, principally bacterial in nature.

Secondly, there are many pathogens, including *S. aureus*, *S. pneumoniae* and *N. meningitidis* to which the entire population is exposed^37^, but from which only a small proportion of subjects develop clinical disease. The basis of natural protection to these pathogens is poorly understood, and UK Biobank and similar cohort studies offer the opportunity to ‘learn from natural protection’, for example by genome wide association studies.

The third opportunity concerns the monitoring of antimicrobial resistance using this platform. There is an expectation that large healthcare databases can be used for continuous monitoring of population health^38^; monitoring the spread of antimicrobial resistance is one example^19,38^. UK Biobank now stores diagnosis and prescribing information from general practice consultations for about half of the cohort up until 2016, through which 75% of the UK’s human antimicrobial exposure occurs^19^, as well as hospital inpatient data for all 500,000 participants. This wealth of data on health outcomes, when combined with data from SGSS, will make it possible to analyse specific outcomes of interest (e.g., death, or hospital admission) associated with a given infection and taking into account co-morbidities, prior antimicrobial exposure (which selects for resistance), and the prior isolation of resistant microbes (which is a measure of resistance in the subject’s flora^5^), and of current antibiotic exposure. Such data will enable research into in vivo antibiotic failure in the general population, a capability which does not currently exist at scale in the UK.

Additionally, new technologies enable identification of bacterial genetic elements and variants causally associated with virulence determinants, including novel antibiotic resistance elements^39–43^. Such approaches have now been applied to a wide range of pathogens^44–49^. As bacterial genome sequencing declines in cost, it has become possible to consider surveillance of pathogen populations being isolated from sites of infection at a genomic level; bacterial loci associated with virulence, spread, or antimicrobial resistance could all be identified by analyses co-modelling antimicrobial exposures and the other comorbidities, and for the identification of human-bacterial genetic interactions^41^. The challenge is that specimen collection mechanisms would have to be established to do this. However, since 50% of the UK Biobank subjects’ samples are accrued in only 12 laboratories, using these laboratories as sentinel sites to gather microbial isolations as they are obtained, together with a program of centralised banking and sequencing, could be considered.

This approach, which is feasible given the record linkage process put in place here, would be globally unique. Inclusion of microbiological endpoints in the UK Biobank will allow this important resource to be used to address a range of important question related to infection in older adults.

## Methods

### Collection of data from microbial laboratories by SGSS

All microbiological laboratories in England send data about identification of microbiological isolates to a central PHE database, the second generation surveillance system. The SGSS dataset is updated on a daily basis via two data feeds. One contains mandatory reporting of a narrow range of pathogens of particular public health importance, including *Salmonella*, *Campylobacter* and other foodborne pathogens. A second data feed includes details of all microbial cultures on which antimicrobial susceptibility testing was performed. The antimicrobial susceptibility results transmitted to SGSS include all antimicrobial tests performed and their results, not just the clinically relevant subset reported by the laboratory to the clinician requesting the test. SGSS performs quality control checks and applies mappings between terms used by individual laboratories (including specimen types and microbiological species) to generate a standardised dataset.

We considered isolates received from individuals resident within English local authorities between 1 April 2010 and 30 June 2016, which we term the *study period*, unless otherwise stated. We made this restriction because coverage of Wales and Scotland by SGSS is not complete. We also restricted the comparative analysis of the general population to individuals of the same age range as that of UKB participants.

### Transfer and storage of data from UK Biobank

To establish which UK Biobank participants were also in SGSS*,* we used a system for encryption and storage of pseudonymised identifiers (OpenPseudonymiser^11^) to compare pseudo-anonymised (tokenised) NHS numbers present on SGSS records with the tokenised NHS numbers of UKB participants. This arrangement allows PHE to identify records from UK Biobank participants, but does not reveal to PHE the identity of the entire UK Biobank cohort. In exploratory analyses, additional identifiers (comprising date of birth, initial of forename and full surname, sex) were similarly tokenised to assess the feasibility of linkage of SGSS entries which lacked NHS numbers.

### Data linkage strategy

We compared record linkage using NHS number alone vs. linkage using additional identifiers (surname, forename, date birth), measuring the proportion of linked records, and how this affected the specificity of linkage, i.e., the proportion of records falsely linked to an individual. To do so, we computed mean number of unique NHS numbers for sets of records putatively belonging to an individual, as identified by various composite identifiers made up of forename, surname, and date of birth. These investigations indicated that use of composite identifiers (in addition to NHS numbers) were likely to link records from individuals other than the intended individual.

### Population estimates and computation of rates

Rates of recruitment and microbial isolation were estimated for each Local Authority (based on the participant’s address at recruitment), even when the address details provided to SGSS indicated they had moved. We used mid-year estimates of the population aged 40–69 resident in local authorities from which UKB recruited, stratified by gender, when comparing isolation rates in UKB subjects and in the general population. This data was obtained from the Office for National Statistics, UK.

### Healthy participant effect

Health outcomes and health-seeking behaviour differ between UK Biobank participants and the general population^33^, with evidence of a “healthy participant” effect. To assess whether such effects also apply to infection outcomes, we compared isolation rates between UK Biobank participants and similarly aged individuals (age 40–69) living in the English local authorities from which the UK Biobank recruited. We stratified isolation rates by gender, and by whether the specimen was received from hospital (secondary care) or from general practitioners (primary care). We computed incidence rate ratios (IRRs) in four population strata: males with isolation from hospital, females with isolation from hospital, males with isolation from outside hospital (e.g. GP), females with isolation from outside hospital. We studied isolation from any site of four groups of organisms: *S. pneumoniae*, *S. aureus*, *E. coli* overall, and *E. coli* with cephalosporine or quinolone resistance, groups chosen because of their medical important. Thus, for each population/organism combination, we constructed a 2 × 2 table representing individuals at risk (i.e. in the relevant population, or in the UK Biobank cohort) but without positive cultures, and those with positive cultures. We computed incidence rate ratios using the epi.2by2 package in the R epiR package for R 3.11.

### Ethics statement

PHE gathers data from NHS microbiology laboratories, storing it in the SGSS database. This data is fully identified, and anonymised extracts are generated prior to epidemiological analysis. This activity is permitted under Section 251 of the National Health Service Act 2006, which allows processing of named patient data without consent for defined purposes, including public health surveillance. Participants in the UK Biobank gave written, informed consent for UK Biobank to follow their health using linkage to electronic health-related records. All methods were carried out in accordance with relevant guidelines and regulations as detailed in the ethical framework used by UK Biobank, which have been published^21^. No experimental protocols requiring prior institutional and/or licencing committee approval were undertaken.

## Supplementary Information

Supplementary Information.

## Data Availability

The code written for database linkage in this study is internal to UK Health Security Agency (UKHSA) systems, and will not be released publicly. It is planned that data provided by the UKHSA system will be incorporated into the UK Biobank (UKB) database and released through the usual UK Biobank governance processes. Work to effect this is ongoing, and UK Biobank registered researchers will be notified in the normal way.
