# Supplementary material for: Laboratory diagnosed microbial infection in English UK Biobank participants in comparison to the general population
Source: Sci Rep. 2023 Jan 10;13:496. doi: 10.1038/s41598-022-20635-4 (PMC9831014; doi:10.1038/s41598-022-20635-4)
Supplement: Supplementary file 1 — Supplementary Information. [file 41598_2022_20635_MOESM1_ESM.docx]

## Supplementary Tables

| **Laboratory** | **Number of UK Biobank participants with samples recorded in SGSS in 2015** | **Cumulative percentage of culture +ve UKB population** |
| --- | --- | --- |
| 1 | 1239 | 6% |
| 2 | 1079 | 12% |
| 3 | 1028 | 17% |
| 4 | 993 | 22% |
| 5 | 968 | 27% |
| 6 | 897 | 31% |
| 7 | 877 | 36% |
| 8 | 782 | 40% |
| 9 | 689 | 43% |
| 10 | 605 | 46% |
| 11 | 571 | 49% |
| 12 | 489 | 52% |
| 13 | 481 | 54% |
| 14 | 459 | 57% |
| 15 | 443 | 59% |
| 16 | 435 | 61% |
| 17 | 422 | 63% |
| 18 | 366 | 65% |
| 19 | 348 | 67% |
| 20 | 334 | 69% |
| 21 | 327 | 70% |
| 22 | 321 | 72% |
| 23 | 282 | 73% |
| 24 | 277 | 75% |

### Table S1: Laboratories by which UK Biobank subjects were frequently recovered

The number of UK Biobank participants with samples recorded in SGSS in 2015, stratified by laboratory processing the sample. Laboratories are ranked by numbers of UKB samples they received; the laboratories recovering 75% of the samples from the UKB participants are shown.

**Supplementary Figures**

**
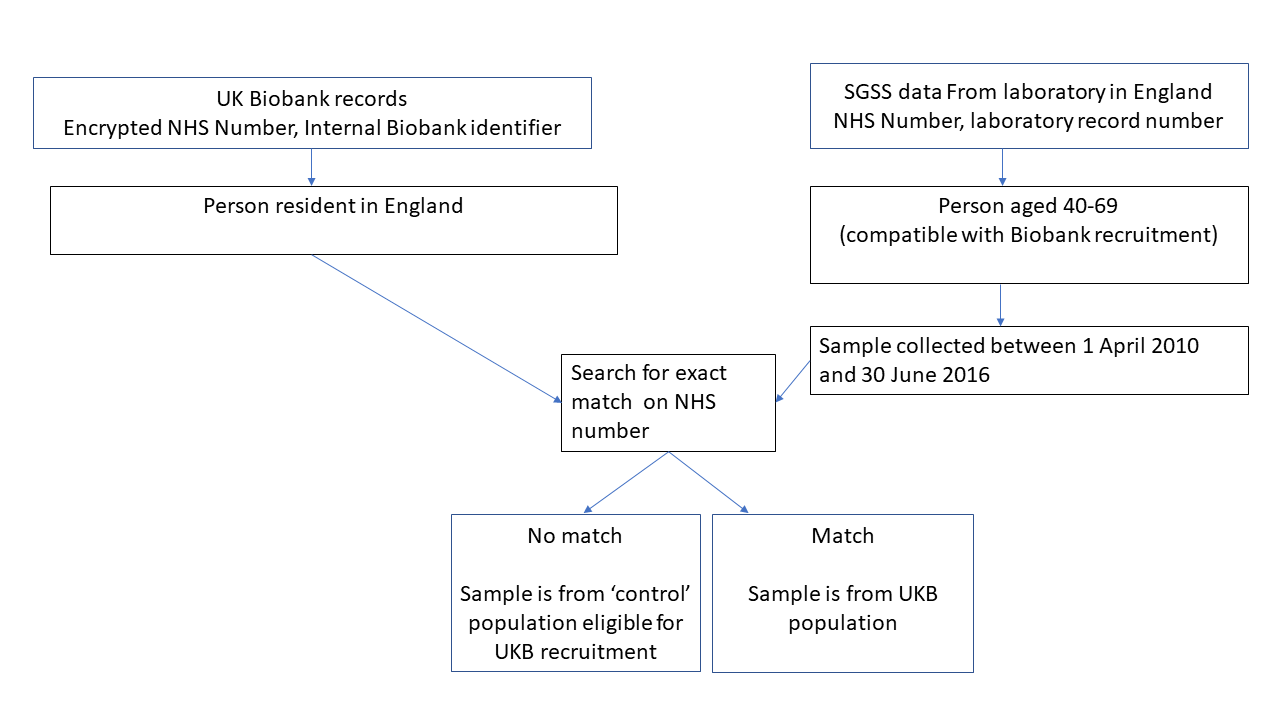
**

**Figure S1 Data linkage diagram**
